# Influencing Factors and Potential Mechanisms of Promoting Postpartum Nurses’ Work Engagement: A Mixed‐Methods Study Based on Conservation of Resources Theory

**DOI:** 10.1155/jonm/5058597

**Published:** 2026-11-01

**Authors:** Keqin Liu, Yang Yang, Suya Li, Qing Yang, Sisi Cai, Mingfeng Yu, Yu Liu

**Affiliations:** ^1^ Department of Nursing Tongji Hospital Tongji Medical College Huazhong University of Science and Technology Wuhan 430030 China hust.edu.cn; ^2^ School of Nursing Tongji Medical College Huazhong University of Science and Technology Wuhan China hust.edu.cn

**Keywords:** mixed methods, nurses, return to work, self-efficacy, social support, work engagement

## Abstract

**Background:**

Returning to work after childbirth represents a critical career transition for nurses, often accompanied by competing role demands and psychological challenges. Work engagement is essential for nurses’ wellbeing and workforce sustainability, yet its underlying associations during the postpartum return‐to‐work period remain poorly understood.

**Objective:**

To examine nurses’ work engagement upon returning to work after childbirth and to assess the possible mediating role of self‐efficacy in the association between perceived social support and work engagement.

**Methods:**

An exploratory sequential mixed‐methods design was employed. This study process consisted of three steps: (i) semistructured interviews with 23 nurses, based on the conservation of resources (COR) theory, were analyzed using thematic analysis to identify key themes and subthemes; (ii) appropriate selection of scales according to the COR theory, qualitative results, and relevant literature; and (iii) a cross‐sectional study to explore the factors and pathways of work engagement of postpartum returning nurses. Finally, a joint display was used to integrate the qualitative and quantitative findings.

**Results:**

The qualitative interviews generated three domains that were identified as major influencing factors, including social support, self‐efficacy, and work engagement. Quantitative findings showed that work engagement among nurses returning to work after childbirth was at a moderate level. Regression analysis identified that social support and self‐efficacy were positively associated with work engagement, and self‐efficacy partially mediated the relationship between social support and engagement.

**Conclusions:**

This study explained the interaction association and pathway of work engagement on the dual resources of postpartum returning nurses. This study also provides an in‐depth analysis of influencing factors. Social support appears to be associated with work engagement indirectly, through its role in fostering self‐efficacy, highlighting the importance of supportive and responsive return‐to‐work environments. These findings underscore the need for nursing management strategies that attend to both the emotional and developmental aspects of nurses’ re‐engagement after childbirth.

**Implication for Nursing Management:**

Nursing managers should regard postpartum return to work as a transitional process requiring both organizational support and psychological resource reconstruction. Supportive return‐to‐work pathways, flexible scheduling, mentoring, and constructive feedback could be considered as potential strategies.

## 1. Introduction

Studies have reported that there are 29.8 million nurses worldwide and have shown that one‐third are women of reproductive age [1, 2]. In China, it is estimated that about 3.39 million nurses are in their prime childbearing years, suggesting that a substantial and growing proportion of these nurses will face the postpartum return to work [1, 3]. Returning to work after childbirth is a significant physical and psychological challenge for nurses. Previous studies have shown that nurses returning to work are exposed to several work‐related influencing factors, which may drain their available resources and affect normal work [4–6]. Despite the physical and psychological pressures, nursing is also very fulfilling. Nurses can protect patients’ safety, alleviate suffering from illness, and improve their quality of life.

In this context, research has shown that autonomy or performance feedback has a favorable effect on work engagement [7–9]. Work engagement is defined as “a positive, fulfilling, work‐related state of mind that is characterized by vigor, dedication, and absorption” [10]. In this context, vigor refers to feeling high energy and mental toughness at work; dedication refers to strong commitment and enthusiasm; and absorption refers to focus on your work and having fun doing it quickly. Unlike job satisfaction (affective evaluation), burnout (negative syndrome), and professional commitment (identity and intention to stay), work engagement emphasizes a positive, fulfilling, and work‐related mindset. When job demands are balanced with available resources, job engagement will increase; when the demands of the work become too heavy, stress and burnout can increase [11]. In the medical field, work engagement is recognized as a key factor in promoting ethical nursing practice to ensure high quality care and positive patient treatment outcomes [12].

### 1.1. Background

Existing studies have shown that the overall level of work engagement of postpartum returning nurses needs to be improved, especially under the background of the implementation of the three‐child policy and the continuous shortage of nursing human resources; ensuring the stability and effectiveness of the core nursing team is facing practical challenges [13]. In this context, timely research on the work engagement of postpartum returning nurses is significant in promoting the construction and development of the nursing workforce. Previous studies on work engagement mostly focused on the general population of nurses or focused on internal factors such as resilience, job satisfaction, and job burnout [14–16]. The special transition stage of postpartum return to work and the external situational factors such as the work environment have been underestimated or neglected. Based on the job demand–resource theory, the work engagement of the nurses is the result of the interaction between personal resources and work environment resources [17]. The hospital is the main place for nurses to realize their professional value, and it is also one of the key factors affecting the adaptation and development of postpartum returning nurses. Existing research suggests that perceived social support can significantly improve work engagement among nurses, such as sense of belonging and vitality [18]. Social support refers to support from social interactions between individuals, groups, or communities [19]. Studies have shown that when social support meets the developmental needs of postpartum returning nurses, it is beneficial to promote their physical and mental health development [20]. When the work atmosphere cannot meet the developmental needs of postpartum returning nurses, it is easy to cause internal and external problems, including psychological problems, job burnout, career adjustment disorders, and even resignation [21, 22].

Quantitative studies further clarify the specific degree of risk of the above problems. In terms of job burnout, He et al. used the Maslach Burnout Inventory (MBI‐GS) to measure the nurses returning to work after the second child [23]. The results showed that the emotional exhaustion score of the control group was 12.59 ± 4.55, the sense of working indifference score was 12.90 ± 5.75, and the personal accomplishment score was 22.16 ± 5.34. Compared with ordinary shift nurses (emotional exhaustion: 9.078 ± 6.07; working indifference: 5.50 ± 4.96; and personal accomplishment: 21.10 ± 9.61), postpartum returning nurses’ emotional exhaustion and sense of cynicism were at an upper‐middle level, suggesting that there was a certain degree of job burnout [24]. Job burnout can diminish the work enthusiasm of nurses, leading to a reduction in the quality of nursing services they provide. In terms of occupational adjustment disorders, Yu et al. showed that the score of work adjustment disorder of postpartum returning nurses was (19.53 ± 0.74), which was higher than that of the Chinese norm (17.28 ± 5.14), indicating that postpartum returning nurses lacked work motivation and had obvious work maladjustment [25, 26]. Further analyzing the impact of work pressure, studies on work pressure show that postpartum returning nurses generally have high work pressure, and turnover intention and mental health problems are the main influencing factors of work pressure, thereby reducing the level of work engagement [27]. Based on the quantitative evidence described above, positive social support is thought to alleviate these problems. Li et al. found that a positive and supportive social atmosphere helps postpartum returning nurses strengthen their identification with the nursing profession to reduce internal and external problems [20, 21]. In the clinical environment, a supportive environment may be a possible explanatory pathway to the work engagement of postpartum returning nurses, which not only directly affects the health outcomes of patients but also improves job satisfaction and nursing quality. Therefore, we propose that social support is closely related to work engagement.

How external factors act on individuals to alter work engagement is unknown and provides a limited reference for targeted intervention strategies. Self‐efficacy, an important concept in work engagement, refers to the confidence in one’s ability to organize and execute actions to achieve specific goals and is an important factor in how individuals manage stress, which can affect an individual’s attitudes, emotions, and behaviors [28]. Kohnen et al. found that work engagement may further affect postpartum nurses’ job satisfaction and career quality, which in turn enhances their professional confidence and potentially increases professional commitment [29]. Meanwhile, previous studies have shown that good social support and job resources can promote nurses’ work motivation and psychological adaptation, and self‐efficacy is the key internal motivation to promote work engagement [30]. According to the conservation of resources (COR) theory, social support can enhance individual self‐efficacy and high self‐efficacy helps individuals achieve better work engagement. Therefore, social support may foster work engagement among postpartum returning nurses by improving self‐efficacy, strengthening healthcare sustainability, and improving patient outcomes.

Most of the prior research has utilized semistructured interviews to explore the perspectives of postpartum returning nurses on work stress and work experiences, resulting in the perspective of work engagement not being fully explored [31, 32]. Additionally, some studies on work engagement have predominantly employed traditional quantitative methods, and there was a lack of targeted investigation on the special group of postpartum returning nurses [9, 14]. However, these approaches are limited by their susceptibility to recall bias and social desirability effects, which can inhibit the capture of spontaneous, unfiltered narratives in complex contexts [33]. In light of the evidence, we hypothesize that work engagement is positively associated with social support and self‐efficacy and that self‐efficacy plays a mediating role between social support and work engagement. However, although quantitative studies can verify the existence of such mediating effects, they cannot explain the underlying associations.

Therefore, this study adopted an exploratory sequential mixed‐methods study considering the complexity and multidimensional nature of work engagement of postpartum nurses. In this study design, the qualitative study first explored the inner experience and subjective feelings of postpartum returning nurses on work engagement based on the COR theory to provide a basis for hypotheses. Subsequently, a quantitative study validated the qualitative findings through data collection and analysis. This complementary and validating research approach allows us to obtain more in‐depth research results. Furthermore, this study aims to comprehensively reveal the internal associations of social support affecting the work engagement of postpartum returning nurses through self‐efficacy.

### 1.2. Theoretical Background

This study is guided by the COR theory model [34]. The COR model, proposed by American psychologist Stevan E. Hobfoll (1989), is concise and highly operable, which makes it suitable for revealing the underlying logic of human responses to environmental challenges by managing limited resources [34]. The model classifies resources into four categories: material resources, energy resources, individual trait resources, and social resources (see Supporting Information Figure 1). According to Hobfoll, social support is regarded as material and energy resources, self‐efficacy as individual trait resources, and work engagement as the result of resource gain. Based on this, a theoretical research hypothesis was put forward, and social support may be a possible explanatory pathway to the work engagement of postpartum returning nurses by enhancing self‐efficacy. Therefore, this study utilized the COR theoretical model as the research framework to guide both quantitative and qualitative research.

Within this framework, perceived social support was regarded as an external social resource, including emotional, instrumental, informational, and appraisal support. Self‐efficacy was regarded as a personal psychological resource reflecting nurses’ confidence in managing clinical tasks and balancing maternal and professional roles. Work engagement was regarded as a positive work‐related state that may emerge when nurses have sufficient resources to invest in their professional role. Based on COR theory, perceived social support may be associated with work engagement both directly and indirectly through self‐efficacy. Therefore, we hypothesized that self‐efficacy would partially mediate the association between perceived social support and work engagement.

## 2. Methods

This study complied with the Strengthening the Reporting of Observational Studies in Epidemiology (STROBE) guidelines for cross‐sectional studies and used the Mixed‐Methods Article Reporting Standards (MMARS) checklist to report the findings (see Supporting Information Table 1) [35].

### 2.1. Study Design

This study adopted an exploratory sequential mixed‐methods design to broadly explore the work engagement experience of postpartum returning nurses and its influencing factors [36, 37]. The study process is divided into four steps (see Figure 1).

**FIGURE 1:**
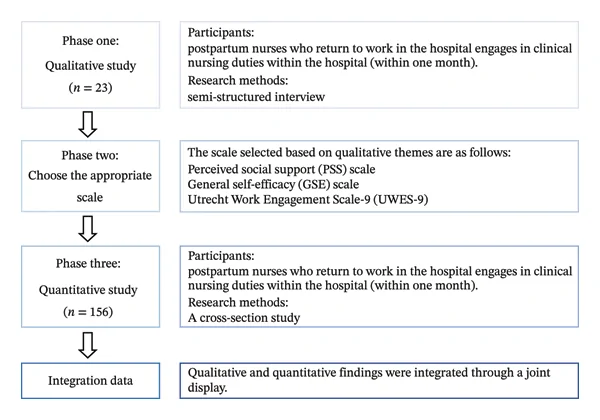
Research framework of the sequential exploratory mixed‐methods study.

### 2.2. Participants and Settings

This study was conducted from March 2025 to November 2025 in three campuses (Hankou, Guanggu and Zhongfa campuses) of a large tertiary general hospital in Hubei Province, China. Although participants were recruited from three campuses to increase sample diversity, all campuses belonged to one tertiary hospital system. Therefore, the findings should be interpreted with caution when generalized to other institutional or regional contexts.

#### 2.2.1. Qualitative Phase

To enhance the range of perspectives represented, purposive sampling was employed to select interested interviewees from various stages of engagement and sociodemographic characteristics as indicated. The inclusion criteria were that participants must be (i) postpartum nurses who return to work in the hospital and engage in clinical nursing duties within the hospital; (ii) the return‐to‐work period after childbirth is within 1 month; and (iii) informed and voluntary participation in this study. Exclusion criteria were individuals with advanced study, rotation, practice nurses, and those unable to understand and complete the study. The sample size was monitored continuously to identify when saturation occurred. After interviewing 23 nurses returning to work after childbirth, we stopped recruitment because the codebook tended to be stable, and no new codes were generated.

#### 2.2.2. Quantitative Phase

Postpartum returning nurses were recruited via convenience sampling. The inclusion and exclusion criteria were the same as those in the qualitative phase. The sample size was calculated to accurately estimate the proportion of nurses returning to work postpartum, using the standard formula for proportion estimation [*n* = *z* ^2^ ∗ *p* ∗ (1 − *p*)/*e* ^2^], based on the following indicators: confidence level = 95%, error range = 5%, population proportion = 5% (from the incidence of postpartum returning nurses in our hospital); the minimum sample size needed was 73 participants to meet the study requirements. This estimate was used only as a preliminary estimate of the proportion of the target population and was not the main power basis for multiple regression and mediation effects. In addition, considering the planned regression and mediation analyses, the final sample size was evaluated according to the commonly recommended rule of at least 10–15 participants per predictor variable in regression models. The final sample size of 156 exceeded this minimum requirement and was considered acceptable for the exploratory quantitative analysis. However, the sample size was not specifically based on the indirect‐effects design. Mediation effects involve indirect paths, which are generally less powered than ordinary regression and may be insufficient for smaller indirect effects.

### 2.3. Data Collection

Collection of data for phase one (qualitative) began from March 10th to 31th, 2025; the analysis was completed in March, 2025. Survey items were constructed based on the formulated meanings and significant statements of the themes and theme clusters. The appropriate scale at March, 2025. Collection of data for phase three (quantitative) began from March to November 2025; the analysis was completed between December 2025 and June 2026. The detailed procedures are presented in Supporting Information Figure 2 and Supporting Information Table 2.

#### 2.3.1. Qualitative Phase

To explore the influencing factors and association of work engagement of postpartum returning nurses, based on the COR theory, the research team worked with experienced qualitative researchers and nursing management experts to formulate an interview outline. This guideline is based on the literature review results of the research team [5, 21]. The topic guides are presented in Supporting Information Table 3.

Qualitative data collection was carried out in a quiet conference room in the hospital using the semistructured interview form. Before the interviews, written informed consent was obtained from eligible nurses. During the interviews, interviewers wrote supplemental notes about various changes in the participants’ facial expressions, body movements, and reflections as far as possible so that the participants’ perspectives were correctly stated and comprehended. Finally, interviews were conducted by graduate nursing students who had no relationship with the participants and, therefore, maintained an objective position during the interviews. Each participant participated in a formal interview lasting between 30 and 60 min. All interviews were recorded using a digital recorder. Two different researchers transcribed each interview upon completion.

#### 2.3.2. Quantitative Phase

Combined with the literature review results of the research team and analysis of qualitative phase data, a general demographic information questionnaire and a questionnaire on work engagement‐related experience were designed. The perceived social support scale, the general self‐efficacy (GSE) scale, and the Utrecht Work Engagement Scale‐9 (UWES‐9) were used [21, 38, 39] (see Supporting Information Table 4).

Two researchers contacted returning postpartum nurses who were interested in participating in this study. After screening for eligibility, the researcher explained the purpose and content of the study. The social support, GSE, and UWES were administered to postpartum returning nurses via an online survey link in the quantitative part of the study. An informed consent form was included in this online platform. It took an average of 15–30 min to complete these forms. Postpartum returning nurses who completed the whole study were provided with sleep support monitoring and a postpartum return gift package provided by the nursing department.

### 2.4. Data Analysis

#### 2.4.1. Qualitative Phase

The qualitative data were analyzed using the inductive thematic analysis approach proposed by Braun and Clarke, which guided this process [40]. Data collection was synchronized with data analysis to determine whether coding had reached data saturation. The first authors (KL and YY), who are registered nurses with multiple years of experience in nursing research, performed the coding. The corresponding author (YL) is a professor of nursing management. Two coders systematically conducted an in‐depth theoretical study of qualitative research and received guidance from experts with rich experience in qualitative research. Meanwhile, extracted data were imported into NVivo software for coding and recorded in Microsoft Excel.

The data analysis process took place in three steps. First, by reading and rereading the transcripts, the researchers immersed themselves in the data to become familiar with the depth and breadth of the content. Second, they engaged in open coding separately in line with the research objectives. Finally, all codes were compared for similarities and differences, and similar codes were summarized into subthemes, which were further reviewed, merged, and refined into candidate themes. Disagreements between the researchers were resolved through group discussion and re‐evaluation to reach a final consensus.

#### 2.4.2. Quantitative Phase

Quantitative data were analyzed using IBM SPSS software, R 4.5.1, and PROCESS macro (Model 4). Measurement data were expressed as mean ± standard deviation, and count data were expressed as frequency (%). Independent *t* tests were used to compare differences in work engagement and scores on each dimension of dichotomous demographic variables; the Mann–Whitney *U* test was used for continuous variables with nonnormal distribution or uneven variance in the above comparisons. Chi‐square tests were used to classify associations between demographic variables and work engagement.

Tolerance (TOL) and variance inflation factor (VIF) were used to assess multicollinearity between independent variables (VIF <  5 and TOL >  0.2). Multiple linear regression was used to analyze the relationship between demographic and characteristics (see Table 1 for all variables) and work engagement. Pearson correlation was calculated to assess the relationship between social support, self‐efficacy, and work engagement. Mediation modeling was used to test whether self‐efficacy mediated the relationship between social support and work engagement; demographic information was controlled for in the analysis of mediators. Statistical significance was assessed through two‐tailed testing, with a threshold of *p* < 0.05.

**TABLE 1:** Differences in sociodemographic characteristics of work engagement (*n* = 156).

Characteristics	All (*n* = 156)	Work engagement	*t*/*F*	*p*
	Number (%)	*M* ± SD		
*Age*			1.039	0.595
< 30	26 (16.6%)	33.38 ± 9.36		
30–35	107 (68.6%)	33.48 ± 10.29		
> 35	23 (14.8%)	34.52 ± 11.51		

*BMI*			5.068	0.167
< 18.5	9 (5.7%)	31.89 ± 15.58		
18.5–24.0	92 (58.9%)	33.26 ± 10.44		
24.0–28.0	51 (32.6%)	34.98 ± 9.17		
> 28.0	4 (2.5%)	28.25 ± 3.20		

*Education*			2.490	0.648
Bachelor’s degree	136 (87.2%)	33.47 ± 10.59		
Master’s degree	20 (12.8%)	34.60 ± 7.90		

*Working years*			0.554	0.758
< 5	5 (3.2%)	35.80 ± 17.38		
5–10	103 (66.0%)	33.98 ± 8.92		
> 10	48 (30.8%)	32.60 ± 12.16		

*Annual household income*			1.393	0.498
< ¥150,000	16 (10.3%)	32.88 ± 8.66		
¥150,000–300000	105 (67.3%)	33.44 ± 10.68		
> ¥300,000	35 (22.4%)	34.49 ± 9.89		

*Department*			3.899	0.564
Internal department	67 (42.9%)	33.39 ± 9.34		
Surgery department	42 (26.9%)	33.83 ± 11.54		
Operating room/anesthesiology department	16 (10.3%)	35.94 ± 11.34		
Emergency and critical care department	4 (2.6%)	34.00 ± 5.48		
Pediatric department	14 (9.0%)	30.29 ± 10.45		
Gynecology and obstetrics	13 (8.3%)	34.69 ± 10.93		

*Professional title*			0.616	0.735
Nurse	3 (1.9%)	32.67 ± 19.63		
Supervisor nurse	95 (60.9%)	33.98 ± 9.98		
Senior nurse	58 (37.2%)	33.07 ± 10.42		

*Mode of delivery*			0.162	0.637
Vaginal delivery	71 (45.5%)	34.04 ± 9.80		
Cesarean section^1^	85 (51.5%)	33.26 ± 10.69		

*Number of deliveries*			2.002	0.146
1	101 (64.7%)	32.73 ± 9.73		
≥ 2	55 (35.3%)	35.24 ± 11.11		

*Feeding patterns*			1.393	0.498
Breastfeeding^2^	30 (19.2%)	33.13 ± 11.03		
Artificial feeding	73 (46.8%)	34.26 ± 11.05		
Mixed feeding^3^	50 (32.1%)	33.38 ± 9.53		

*Health status*			13.002	0.002^∗∗^
Poor	6 (3.8%)	28.33 ± 11.40		
Ordinary	86 (55.1%)	31.34 ± 10.04		
Fitness	64 (41.0%)	37.17 ± 9.52		

^1^Cesarean section included emergency cesarean section and elective cesarean section.

^2^Breastfeeding included exclusive breastfeeding (direct breastfeeding) and exclusive breastfeeding (direct breastfeeding/bottle feeding).

^3^Mixed feeding included mixed feeding (direct breastfeeding) and mixed feeding (direct breastfeeding/bottle feeding).

^∗^ *p* < 0.05.

^∗∗^ *p* < 0.01.

^∗∗∗^ *p* < 0.001.

#### 2.4.3. Integration of Qualitative and Quantitative Data

To enhance the rigor and transparency of the study, multiple approaches were used to integrate qualitative and quantitative data. First, the results from the initial qualitative phase were helpful in identifying the influencing factors in the quantitative phase; namely, the “follow the cue approach” was applied [5, 20]. Second, the two data types were analyzed separately, and data triangulation enabled the identification of similarities and differences between qualitative and quantitative data. Finally, by integrating the qualitative and quantitative data, the findings elaborated or clarified each other based on the three patterns of “confirmation, elaboration, and dissonance.” In this study, we employed a tabular format to enable the parallel presentation of quantitative and qualitative data and findings (see Table 2) [37].

**TABLE 2:** Joint display of quantitative and qualitative research results.

Results of quantitative		Results of qualitative			Relationship between Qual and Quant
Item contents	Score	Theme (qualitative)	Subtheme (qualitative)	Quotation	
Social support	74.51 ± 11.48	Immediate resource effects of social support	Emotional support was a catalyst for work engagement	N3: “My colleague told me that it didn’t matter, I was there.”	Qual explained the Quant
Emotional support	15.78 ± 3.07				
N15: “Even if I am now half of the labor force, the department has not abandoned me.”					
Instrumental support	21.19 ± 3.52		Instrumental support is a supportive resource for work engagement	N3: “Don’t go there. After you have breastfed, I’ll go and finish it for you.”	
Informational support	20.65 ± 3.90		Information support is an empowering resource for work engagement	N1: “Before returning to the work, the head nurse will send me the latest study materials.”	
				N9: “My colleague gave me a guide to return to work, so that I could adapt to the work faster.”	
Appraisal support	16.89 ± 2.87		Appraisal support is a positive feedback resource for work engagement	N12: “Return to work examination not only passed, the performance is very good, it seems that the vacation did not completely lose the effort.”	

Self‐efficacy	28.19 ± 6.44	Fluctuations and reconstruction of self‐efficacy	Self‐doubt at the beginning of returning to work	N3: “The first week or two will be particularly uncomfortable, a little down, and sometimes a little ready to quit.”	Qual explained the Quant
			Confidence rebound from successful experiences	N8: “I feel that I can slowly adapt to it, there may be a process of transformation.”	

Work engagement	33.62 ± 10.27	Cognitive reconstruction of work engagement	The meaning of work engagement	N9: “Work engagement is working without any external interference.”	Quant quantifies Qual
				N10: “I understand work engagement as time and effort invested.”	
				N20: “I understand work engagement as being fully committed to work after going to work.”	
Vigor	10.46 ± 3.66		Value anchors for work engagement	N3: “Constantly gain recognition from the outside world in your work to enhance your inner energy.”	
Dedication	11.68 ± 3.53			N6: “I am not only a mother, but also a professional nurse.”	Qual explained the Quant
Absorption	11.48 ± 3.71			N11: “When I came back to work, I couldn’t get distracted from doing two things.”	

*Note*. Quant = quantitative; Qual = qualitative.

### 2.5. Reflexivity and Trustworthiness

The credibility of this exploratory sequential interpretation of mixed‐methods research was achieved through a meticulous strategy tailored to both qualitative and quantitative aspects. To increase the credibility of this study, the analyses were performed by the first author in repeated dialog with the research team, until agreement was reached on procedures, content, and outcomes. Additionally, the use of standard questionnaires such as social support, GSE, and UWES for the survey ensured validity was accounted for. Meanwhile, the rich narrative enhances the transferability. To improve reliability and confirmability, the original Chinese language was used for data analysis. Two bilingual researchers translated the final themes into English, and the professors in the fields of psychology and management reviewed them. Finally, rigor is ensured through credibility, transferability, reliability, and confirmability.

### 2.6. Ethical Considerations

This study has been approved by the Ethics committee of Tongji Hospital, Tongji Medical College, Huazhong University of Science and Technology (TJ‐IRB202503043). Written or electronic informed consent was obtained from all participants before interviews or questionnaire completion. Participants were informed of the study purpose, voluntary nature of participation, confidentiality of data, and their right to withdraw at any time without penalty. All data were anonymized and used only for research purposes.

## 3. Results

### 3.1. Participant Characteristics

#### 3.1.1. Qualitative Phase

Twenty‐three participants clarified their perceived social support and self‐efficacy on work engagement among postpartum nurses returning to work and reasons for their decisions regarding participation through interviews. Most interviewees were 30–35 years old (18, 78.26%), with a BMI between 14 and 28 and a bachelor’s degree (20, 86.96%). Sixteen interviewees reported working for 5–10 years; the majority were senior nurses (12, 52.17%). Participant demographic characteristics are shown in Table 3.

**TABLE 3:** Characteristics of participants in the qualitative and quantitative stages.

Characteristics	Qualitative stage (*n* = 23)		Quantitative stage (*n* = 156)	
	Number	Proportion (%)	Number	Proportion (%)
*Age*				
< 30	3	13.0	26	16.57
30–35	18	78.3	107	68.59
> 35	2	8.7	23	14.74

*BMI*				
< 18.5	2	8.7	9	5.7
18.5–24.0	10	43.5	92	58.9
24.0–28.0	11	47.8	51	32.6
> 28.0	0	0.00	4	2.5

*Education*				
Bachelor’s degree	20	87.0	136	87.2
Master’s degree	3	13.0	20	12.8

*Working years*				
< 5	1	4.4	5	3.2
5–10	16	69.6	103	66.0
> 10	6	26.1	48	30.8

*Annual household income*				
< ¥150,000	1	4.3	16	10.3
¥150,000–300000	14	60.9	105	67.3
> ¥300,000	8	34.8	35	22.4

*Department*				
Internal department	10	43.5	67	42.9
Surgery department	5	21.7	42	26.9
Operating room/anesthesiology department	2	8.7	16	10.3
Emergency and critical care department	5	21.7	4	2.6
Pediatric department	1	4.4	14	9.0
Gynecology and obstetrics	1	4.4	13	8.3

*Professional title*				
Nurse	0	0	3	1.9
Supervisor nurse	11	47.8	95	60.9
Senior nurse	12	52.2	58	37.2

*Mode of delivery*				
Vaginal delivery	11	47.8	71	45.5
Cesarean Section^1^	12	52.2	85	51.5

*Number of deliveries*				
1	16	69.6	101	64.7
≥ 2	7	30.4	55	35.3

*Feeding patterns*				
Breastfeeding^2^	4	17.4	30	19.2
Artificial feeding	8	34.8	73	46.8
Mixed feeding^3^	11	47.8	50	32.1

*Health status*				
Poor	4	17.4	6	3.8
Ordinary	9	39.1	86	55.1
Fitness	10	43.8	64	41.0

^1^Cesarean section included emergency cesarean section and elective cesarean section.

^2^Breastfeeding included exclusive breastfeeding (direct breastfeeding) and exclusive breastfeeding (direct breastfeeding/bottle feeding).

^3^Mixed feeding included mixed feeding (direct breastfeeding) and mixed feeding (direct breastfeeding/bottle feeding).

#### 3.1.2. Quantitative Phase

The mean age of the participants was 32.19 years (SD = 3.00, range = 21–42). The mean height was 162.56 cm (SD = 4.35, range = 151–178), and the mean body weight was 60.61 kg (SD = 8.55, range = 42–116). The majority of participants had 5–10 years of work experience (66.0%). More detailed participant characteristics are presented in Table 3.

### 3.2. Qualitative Results

When organizing the data from participants’ responses, three main categories and eight subcategories were identified (see Table 2).

#### 3.2.1. Immediate Resource Effects of Social Support

##### 3.2.1.1. Emotional Support Was a Catalyst for Work Engagement

When asked how to actively identify or accept emotional care from others during the process of returning to work, some participants said they were relatively unfamiliar with actively perceiving and expressing “emotional support,” and they were rarely aware that they needed or could openly seek such understanding and comfort.

N2: If you return to work, it is mainly your psychological preparation! I think this is too mentally ready; I don’t think anything else is physically possible, and I think it can be overcome.

N20: Just back to work, I am very stressed, a little helpless, and then I slowly adjust my mood, and now I can be better.

##### 3.2.1.2. Instrumental Support Is a Supportive Resource for Work Engagement

When asked to share specific, practical work or life support they have received since returning to work, some participants said they received ample instrumental support.

N7: For example, the nursing department will issue postpartum return gift packages, and the nursing department will have some follow‐up activities that are presented and implemented in different forms to support our better return to work.

N18: In the past period (when I just returned to work), you would not be allowed to work alone, and there would be colleagues with you. At that time, because I was basically on a day shift, each shift would have a separate person to take me.

##### 3.2.1.3. Information Support Is an Empowering Resource for Work Engagement

Participants expressed that effective information support was a unique cognitive navigation experience, which was a process of constant coordination with new roles and new rhythms. It helped them to sort out physical and mental changes and job requirements, integrate professional guidance and personal experience, and establish a feasible balance between motherhood and professional roles by obtaining timely and accurate knowledge and advice.

N12: The day before I went to work, I went to the department in advance to have a little understanding of the rhythm and changes of the department, and my colleagues would take the initiative to explain to me any unfamiliar places.

N22: Colleagues and leaders of the department will share some learning materials in advance, so that I can also submit to learn the new business and new technology of the department, and gradually get familiar with and adapt to the pace of work.

##### 3.2.1.4. Appraisal Support Is a Positive Feedback Resource for Work Engagement

Feeling recognized by your colleagues and team cannot be taken for granted. Many participants valued and appreciated the professional trust, positive feedback, and validation of their ability to work that they had received since returning to work. In addition, they also view the challenges and adjustments encountered in the early stage of returning to work as a special process of career growth and role integration and view this transition with a more accepting attitude.

N1: When I first started working, my colleagues took good care of me. For example, I worked very slowly, sometimes not very well; they were very enthusiastic about teaching me. The leader also took good care of me.

N3: The working environment will affect my working state, but my colleagues and leaders take good care of me and let me quickly adapt to the pace of work.

#### 3.2.2. Fluctuations and Reconstruction of Self‐Efficacy

##### 3.2.2.1. Role Transition Uncertainty and Professional Self‐Doubt

Many of the participants interviewed spoke of regaining their sense of professional value by returning to the clinic and successfully caring for patients. Although they experienced strong tension and self‐doubt between family and work in the early stage of returning to work, they found that when they were no longer tangled in “perfection” but focused on achieving a smooth landing of professional ability in each moment, they could gain more solid confidence.

N4: Actually, go back to the room and take care of the patient. I just feel that professional me, back a little bit.

N9: After that, don’t force yourself to go back to the state before giving birth; today, more than yesterday, do a little more, a little better, take your time.

##### 3.2.2.2. Confidence Rebound From Successful Experiences

There was a general expectation of a “smooth transition” among participants. This does not mean avoiding challenges, but it means that they need to accept their dual new identities as “nurses” and “mothers” cognitively, allow themselves a reasonable adaptation period emotionally, and autonomously regulate the rhythm of work and family to a certain extent, to rebuild the sense of control and efficacy of work in a self‐controlled way.

N3: Between the two roles of nurse and mother, find my own rhythm to switch; this is the best transition.

N16: Because the leadership may also consider that you have just started, they will not give you the kind of very important, relatively high‐pressure job. Then, slowly you move into a working state; the ensuing pressure will come. I hope to take my time.

#### 3.2.3. Cognitive Reconstruction of Work Engagement

This theme describes the understanding of work engagement, which appeared to vary from the participants’ perspectives. Most postpartum returning nurses found work engagement unclear in its meaning and values.

##### 3.2.3.1. The Meaning of Work Engagement

The connotation of work engagement has been redefined in the discourse system of postpartum returning nurses. It is evolving from a “quantifiable effort of physical strength and time” to a “deep commitment of professional presence.”

N8: I understand that work engagement is to ensure that the class can be good, for a positive state of work.

N15: Work engagement, I think, is a time and energy input. Then see how much of your day is devoted to work, and how your physical and mental state can adapt to the intensity of the work.

N21: I think work engagement is “choose what I love, love what I choose.”

##### 3.2.3.2. Value Anchors for Work Engagement

Dedicated postpartum returning nurses are usually full of enthusiasm for their work, feel the meaning and value of their work, and get a sense of achievement and pride from it. Meanwhile, dedicated postpartum returning nurses are usually fully engaged in their work tasks, which is also reflected in their deep participation and enjoyment of work.

N4: I was no longer just a “novice in need of care” I was a professional nurse who could solve clinical problems. I definitely treat patients with a very focused and serious attitude to treat them.

N15: The biggest change was that I began to work independently. Postpartum, going back to work began in the ward; my heart always worried and missed the baby. Now, once I′m in the operational or evaluative state, I can automatically shut out those anxieties for a while and see only the patient and the task at hand.

### 3.3. Quantitative Results

#### 3.3.1. Sociodemographic Characteristics of Participants

The range of scores for the total work engagement scale was 0–54, and the mean work engagement score was 33.62 (SD = 10.27); the average score of the items was 3.74 (SD = 1.14) at a moderate level (2.89–4.66). The scores of each dimension from high to low were dedication (mean: 11.68, SD: 3.53), vigor (mean: 10.46, SD: 3.66), and absorption (mean: 11.48, SD: 3.71); the range of scores for each subscale item was 0–18. The range of scores for the total social support scale was 18–90, and the mean social support score was 74.51 (SD = 11.48). The scores of each dimension from high to low were instrumental support (mean: 21.19, SD: 3.52), informational support (mean: 20.65, SD: 3.90), appraisal support (mean: 16.89, SD: 2.87), and emotional support (mean: 15.78, SD: 3.07). The range of scores for the self‐efficacy scale was 10–40, and the mean self‐efficacy score was 28.19 (SD = 6.44). The results are shown in Supporting Information Table 5 and Supporting Information Figure 3.

#### 3.3.2. Univariate/Multivariate Analysis of Factors Associated With Work Engagement

In univariate analysis, we found that health status had a statistically significant effect on work engagement. Among 156 nurses returning to work after childbirth, poor (3.8%), ordinary (55.1%), and fit (41.0%), with statistical differences between groups (*p* = 0.002). Table 1 describes the differences in demographic characteristics of work engagement. Multiple linear regression analysis was conducted to examine factors associated with work engagement. The results are shown in Table 4. The results were statistically significant in the regression analysis (*R* ^2^ = 0.582, *F* = 15.183, *p* < 0.001), and the explanatory power of the model was 54.3%. Work engagement was significantly affected by social support (*β* = 0.532, *p* < 0.001) and self‐efficacy (*β* = 0.524, *p* < 0.001).

**TABLE 4:** Multiple linear regression analysis of work engagement in postpartum returning nurses.

Variable	*B*	Work engagement						
		Se	*β*	*t*	*p*	95% CI	TOL	VIF
Constant	−16.223	7.710	—	−2.104	0.037	−31.465∼−0.981	—	—
Social support	0.475	0.063	0.532	7.512	< 0.001	0.350–0.600	0.588	1.700
Self‐efficacy	0.524	0.115	0.328	4.542	< 0.001	0.296–0.752	0.565	1.771

*Note:* Only variables with *p* < 0.05 were showed; *R* ^2^ = 0.582, adjusted *R* ^2^ = 0.543, *F* = 15.183. *B* = unstandardized regression coefficient; *β = *standardized regression coefficient.

Abbreviations: CI, confidence interval; TOR, tolerance; VIF, variance inflation factor.

#### 3.3.3. Correlation Between Social Support, Self‐Efficacy, and Work Engagement

Work engagement was positively correlated with social support (*r* = 0.702, *p* < 0.01) and self‐efficacy (*r* = 0.671, *p* < 0.01). In addition, social support was positively correlated with self‐efficacy (*r* = 0.591, *p* < 0.01). The correlation analysis results are presented in Figure 2. Meanwhile, the analysis showed that self‐efficacy played a mediating role between social support and work engagement (see Figure 3). Perceived social support was positively associated with self‐efficacy (*a* = 0.331, *p* < 0.001), and self‐efficacy was positively associated with work engagement after controlling for perceived social support (*b* = 0.497, *p* < 0.001). The total effect of perceived social support on work engagement was significant (*c* = 0.627, *p* < 0.001), and the direct effect remained significant after self‐efficacy was included in the model (*c* ^′^ = 0.463, *p* < 0.001). The indirect effect was significant (ab = 0.165, 95% bootstrap CI: 0.087–0.260), indicating a possible partial mediating pathway.

**FIGURE 2:**
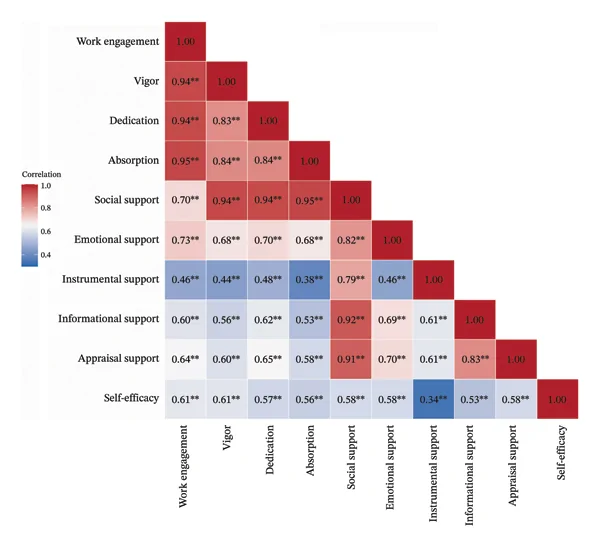
Correlation heatmap of key variables. ^∗^ *p* < 0.05; ^∗∗^ *p* < 0.01.

**FIGURE 3:**
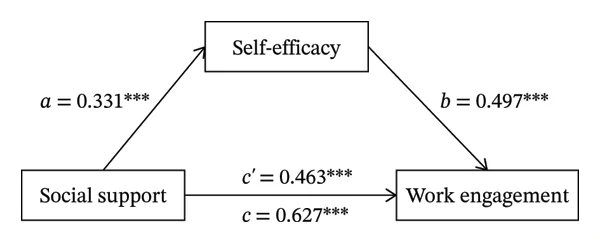
Mediation model of social support, self‐efficacy, and work engagement among postpartum return‐to‐work nurses (unstandardized coefficients). ^∗∗∗^ *p* < 0.001.

### 3.4. Findings From Integration of Qualitative and Quantitative Results

The integration of qualitative and quantitative findings generated a resource‐based interpretation of work engagement among nurses returning to work after childbirth (see Figure 4). A joint display of the qualitative themes and quantitative findings is presented in Table 2, facilitating a comprehensive understanding of work engagement among postpartum nurses.

**FIGURE 4:**
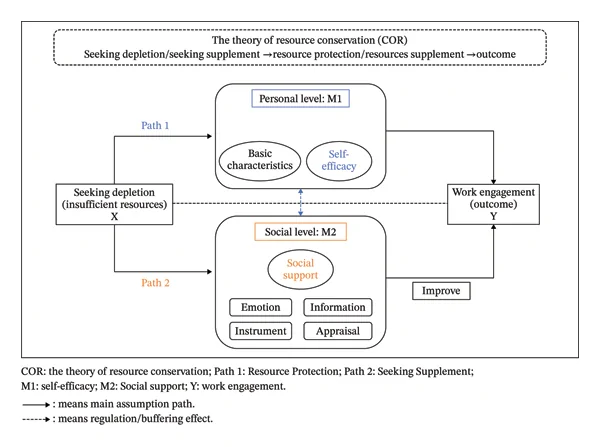
The main framework diagram based on the COR theory. COR: the theory of resource conservation; Path 1: resource protection; Path 2: seeking supplement; M1: self‐efficacy; M2: social support; Y: work engagement. 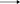: means main assumption path. 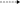: means regulation/buffering effect.

Qualitative research reveals the psychological transformation process of postpartum returning nurses from “self‐doubt” to “confidence reconstruction.” In the early stage of returning to work, nurses experienced a strong sense of incompetence due to lagging skills, role conflicts, and separation from their infants, which manifested as a temporary weakening of their self‐efficacy. Over time, they rebuilt their sense of efficacy through three paths: (i) proficient clinical operation experience, (ii) supportive feedback from colleagues and managers, and (iii) self‐adaptation of work engagement. These three factors add up to push nurses out of self‐doubt.

The qualitative findings provide a mechanistic explanation for “self‐efficacy partially mediating the relationship between perceived social support and work engagement” in the quantitative study. Meanwhile, this complemented the quantitative finding that self‐efficacy was positively associated with work engagement and partially mediated the association between perceived social support and work engagement. Additionally, qualitative findings further revealed the specific connotation of the mediating path: external social support did not directly improve work engagement, but indirectly played a role by helping nurses overcome the trough of “self‐doubt” and rebuild their sense of efficacy.

Overall, the integrated findings suggest a possible resource‐conversion pathway: external social support may help postpartum returning nurses rebuild self‐efficacy, which is in turn associated with higher work engagement.

## 4. Discussion

Using an exploratory sequential mixed‐methods design, this study examined work engagement among nurses returning to work after childbirth and explored the psychological and contextual associations underlying this process. The findings indicate that work engagement among postpartum nurses was at a moderate level and was positively associated with perceived social support and self‐efficacy, with self‐efficacy partially mediating the association between perceived social support and work engagement. These results suggest that postpartum return to work should be understood as a critical career transition rather than a simple continuation of professional practice, during which work engagement may be particularly sensitive to both external resources and internal psychological adaptation.

In this study, UWES‐9 was used to measure work engagement, which contained three dimensions: vigor, dedication, and absorption. The total score of work engagement of postpartum returning nurses was 33.62 ± 10.27, and the total average score was 3.74 ± 1.14, which was at a medium level. Meanwhile, the moderate level of work engagement observed in this study is comparable to the results of studies on the work engagement of clinical nurses in China (33.31 ± 8.22), but it is numerically lower than the work engagement of specialized cancer nurses (47.81 ± 10.62) [41–43]. The consistency of the work engagement results of postpartum returning nurses showed that the work environment introduces unique challenges that may constrain nurses’ capacity to fully invest in their work [31]. However, when compared with nurses in the specialized cancer hospitals without recent childbirth experience, the engagement in our sample was notably lower, reinforcing the notion that temporal life‐stage transitions have an indirect association with work engagement rather than professional role alone. Physical recovery, childcare responsibilities, and concerns regarding infant well‐being coexist with professional expectations, creating a complex environment in which engagement is neither absent nor fully restored [31]. This finding reflects a realistic adaptation process rather than low work engagement. Meanwhile, the importance of viewing work engagement as a dynamic and context‐dependent state rather than a stable individual attribute is emphasized.

Perceived social support was significantly associated with work engagement after controlling for relevant background factors. While this finding aligns with existing evidence based on the Job Demands–Resources framework, the present study extends prior research by suggesting that social support may play a broader role in the postpartum return‐to‐work phase [44]. For nurses navigating dual roles as caregivers and professionals, social support may not merely provide instrumental assistance or informational guidance but may also convey recognition and validation of their multiple identities [21]. Such recognition may help reduce role conflict and emotional strain, thereby creating psychological conditions that enable sustained engagement during a vulnerable transition period. Given that instrumental support was the highest mean social support dimension and that our results revealed persistent gaps in organizational responsiveness, we propose three specific strategies to strengthen social support in managing work engagement. First, structured peer support programs integrate emotional support with work. Second, flexible time arrangements to take care of children’s needs and reduce physical fatigue. Third, staged return‐to‐work competency training to enhance self‐efficacy during the critical return‐to‐work window.

Importantly, self‐efficacy was identified as a partial mediator between perceived social support and work engagement, offering a possible explanation for how external resources may be linked to positive work‐related states. This finding is consistent with social cognitive theory and suggests that social support may be associated with nurses’ confidence in managing clinical tasks, balancing work–family demands, and coping with uncertainty after returning to work [45]. This is similar to Liu’s research findings [31]. At the same time, the partial mediation indicates that perceived social support remained directly associated with work engagement after self‐efficacy was included in the model, suggesting the possibility of additional explanatory pathways, such as emotional reassurance and strengthened professional identity. Together, these results underscore the multifaceted nature of work engagement during postpartum transition and highlight self‐efficacy as a key psychological resource that may link contextual support with work‐related adaptation [46].

The qualitative findings deepen understanding of these quantitative relationships by illuminating how work engagement is experienced in everyday practice. Nurses described persistent concerns about infant health, feelings of guilt associated with mother–infant separation, and emotional tension between maternal and professional roles. These experiences suggest that work engagement in postpartum nurses is embedded in emotional labor and identity negotiation rather than solely reflecting motivation or task involvement [47]. The frequent emphasis on the need for empathy and understanding from supervisors and colleagues further indicates that emotional support may be particularly salient yet insufficiently addressed within existing organizational structures. Research indicates that the mental and physical well‐being of working women returning to work after maternity leave is associated with stronger support from colleagues and supervisors [6, 47].

Qualitative data also shed light on the dynamic nature of self‐efficacy during the return‐to‐work process. Many nurses reported reduced confidence in their clinical competence following time away from practice, particularly when opportunities for gradual skill reacquisition, feedback, and guided supervision were limited. Research on emergency nurses in the United States similarly indicates that re‐entry training is necessary when taking maternity leave and returning to work [48]. This suggests that self‐efficacy in the postpartum period is not a fixed personal resource but a malleable construct shaped by learning experiences, social feedback, and organizational responsiveness [49]. From this perspective, fluctuations in work engagement may reflect broader processes of professional readaptation and identity reconstruction rather than individual motivation alone.

From the perspective of the COR theory, the integrated findings suggest a possible resource‐based explanatory pathway. Postpartum returning nurses may experience resource loss due to physical recovery, childcare responsibilities, work–family role conflict, and professional readaptation. Perceived social support may serve as an external social resource, while self‐efficacy may function as an internal psychological resource. The quantitative findings showed that both resources were associated with work engagement, and the qualitative findings further illustrated how external support may help nurses rebuild confidence and gradually re‐engage in clinical work. However, given the cross‐sectional nature of the quantitative phase, this pathway should be interpreted as a possible explanatory framework rather than evidence of causal mediation.

Several limitations should be acknowledged. First, the quantitative component was conducted in a single institution, which may limit the generalization of the findings to other organizational or cultural contexts. Second, the sample size was not sufficient for indirect effect estimation, and the test power of the mediation path was limited. Small indirect effects may be missed, and the results need to be interpreted with caution. Third, we did not control for mental health variables and objective workload confounders, which may independently deplete nurses’ resources and confound the effects of social support. Finally, although the qualitative findings provided rich insights into nurses’ experiences, variations related to clinical settings, family support structures, and infant health were not explored in depth and warrant further investigation.

## 5. Conclusion

This mixed‐methods study suggests that perceived social support and self‐efficacy are important resources associated with work engagement among nurses returning to work after childbirth. Self‐efficacy partially mediated the association between perceived social support and work engagement, indicating a possible resource‐based explanatory pathway rather than a confirmed causal association. The qualitative findings underscore that early return is a complex transition marked by competing role expectations, physiological strain, and uncertainty and that engagement may be shaped by the degree to which workplaces provide not only empathy and flexibility but also structured opportunities to rebuild competence and confidence. Designing return‐to‐work pathways that explicitly enhance social support and self‐efficacy may be a practical and high‐impact strategy for nursing managers seeking to stabilize the postpartum nursing workforce and sustain high‐quality care.

## Author Contributions

Keqin Liu and Yang Yang contributed to collate and analyze the data and draft and revise the manuscript according to the GRAMMS checklist. Suya Li, Qing Yang, and Sisi Cai are responsible for data collection and processing. Mingfeng Yu and Yu Liu contributed to define the research theme, analyze the data and interpret the results, and revise the manuscript and was responsible for the layout and use of funds.

## Funding

This work was supported by the Hospital fund of Tongji Hospital affiliated to Tongji Medical College of Huazhong University of Science and Technology (2024C06). The funder contributed to defining the research theme, was fully accountable for ensuring the integrity and accuracy of the work, and analyzed the data and interpret the results.

## Disclosure

All the authors have read and approved the final manuscript and reported no declarations of interest.

## Ethics Statement

This study has been approved by the Ethics committee of Tongji Hospital, Tongji Medical College, Huazhong University of Science and Technology (TJ‐IRB202503043, approval date: 10 March 2025).

## Conflicts of Interest

The authors declare no conflicts of interest.

## Supporting Information

Additional supporting information can be found online in the Supporting Information section.

## Supporting information


**Supporting Information** The following supporting information is provided with this article: Supporting Information Figure 1: Conservation of resources theoretical model. Supporting Information Figure 2: The sequential exploratory mixed methods process of this study. Supporting Information Figure 3: Scores on the respective scales (Quantitative). Supporting Information Table 1: Good Reporting of a Mixed Methods Study (GRAMMS) Checklist. Supporting Information Table 2: The specific process of exploratory sequential mixed study. Supporting Information Table 3: Interview Guidelines (Qualitative). Supporting Information Table 4: Introduction to the scales and questionnaires (Quantitative). Supporting Information Table 5: Work engagement scores for postpartum returning nurses (Quantitative).

## Data Availability

The data that support the findings of this study are available on request from the corresponding authors. The data are not publicly available due to privacy or ethical restrictions.
