# Supplementary material for: Influencing Factors and Potential Mechanisms of Promoting Postpartum Nurses’ Work Engagement: A Mixed‐Methods Study Based on Conservation of Resources Theory
Source: J Nurs Manag. 2026 Nov 1;2026:5058597. doi: 10.1155/jonm/5058597 (PMC13725075; doi:10.1155/jonm/5058597)
Supplement: Supplementary file 1 [file JONM-2026-5058597-s001.docx]

**Supporting Information**

**Supporting Information Figure 1**: Conservation of resources theoretical model.

**Supporting Information Figure 2:** The sequential exploratory mixed methods process of this study.

**Supporting Information Figure 3:** Scores on the respective scales (Quantitative).

**Supporting Information Table 1:** Good Reporting of a Mixed Methods Study (GRAMMS) Checklist

**Supporting Information Table 2**: The specific process of exploratory sequential mixed study.

**Supporting Information Table 3**: Interview Guidelines (Qualitative).

**Supporting Information Table 4**: Introduction to the scales and questionnaires (Quantitative).

**Supporting Information Table 5**: Work engagement scores for postpartum returning nurses (Quantitative).

**Supporting Information Figure 1:** Conservation of resources theoretical model.

**Supporting Information Figure 2**: The sequential exploratory mixed methods process of this study.

This figure illustrates the sequential exploratory mixed methods approach, including qualitative and quantitative phases, and the integration of findings. The qualitative data and analysis provide deeper insights by exploring postpartum returning nurses’ perspectives more deeply, identify barriers and facilitators at the early stage of postpartum return to work. The quantitative data and analysis provide a broad understanding of the purpose of a study. Data analysis outputs were integrated through comparative interpretation.

**Supporting Information Figure 3**: Work engagement scores for postpartum returning nurses (Quantitative).

A: I feel energized at work; B. Although the work is stressful at times, I feel happy both physically and mentally; C. When I am immersed in my work, I tend to lose track of time; D. I feel energetic at work; E. I will reach a state of self-denial in my work; F. I’m proud of what I do; G. My work inspires me; H. I am passionate about my work; I. As soon as I got up in the morning, I wanted to go to work in the hospital.

**Supporting Information Table 1:** Good Reporting of a Mixed Methods Study (GRAMMS) Checklist.

| **GUIDELINE** | **Reported on Page No:** |
| --- | --- |
| Describe the justification for using mixed methods approach to the research question | **P6-P7** |
| Describe the design in terms of purpose, priority and sequence of the methods | **P6-P11** |
| Describe each method in terms of sampling, data collection and analysis | **P6-P11** |
| Describe where integration has occurred, how it has occurred and who has participated in it | **P6-P11** |
| Describe any limitation of one method associated with the present of the other method | **P6-P23** |
| Describe any insights gained from mixing or integrating methods | **P22-P23** |

**Supporting Information Table 2**: The specific process of exploratory sequential mixed study.

| Time | Phase | Context | COR theory | literature | Qualitative results | Outcome |
| --- | --- | --- | --- | --- | --- | --- |
| 2025.01 | Theoretical preparation | COR theory | T-CaST | √ |  | Initial framework |
| 2025.03 | **Phase one** | **Qualitative interview** |  |  |  | Theme and sub-themes |
| 2025.03 |  | Data analysis |  |  |  |  |
| 2025.03 | **Phase two** | **Choose the appropriate scale** |  |  |  |  |
|  |  | General Information |  | √ |  |  |
|  |  | Social Support Scale | External resources |  | Specific resources |  |
|  |  | Self-efficacy Scale | Individual resources |  | Personal characteristics resources |  |
|  |  | Work Engagement Scale | Resources accumulation |  | Vigor, dedication, absorption |  |
| 2025.03 |  | Pilot study and expert consultation |  |  |  |  |
| 2025.03-11 | **Phase three** | **A cross-sectional survey** |  |  |  | Formal questionnaire |
| 2025.11-2026.06 |  | Data analysis |  |  |  | Conclusion |
|  |  | Joint display |  |  |  |  |

T-CaST: theory comparison and selection tool; COR theory: conservation of resources theory

**Supporting Information Table 3**: Interview Guidelines (Qualitative).

| **Topic** | **Details** |  |  |  |  |
| --- | --- | --- | --- | --- | --- |
| 1 | How did you feel after returning to work after giving birth, and how much were you engaged in your work? | | | | |
| 2 | How have you been feeling since returning to work after giving birth? | | | | |
| 3 | Which factors do you think would affect your state of work engagement? Which factor do you think has the greatest impact on your work engagement, and why? | | | | |
| 4 | Have you encountered any difficulties or challenges when returning to work after giving birth? | | | | |
| 5 | Did you receive any support or assistance when returning to work after giving birth? What kind of support and assistance do you expect to receive? | | | | |
| 6 | How is your mental health status after giving birth (such as anxiety, depression, stress, and sleep conditions)? Do you think these factors have an impact on your work engagement? | | | | |
| 7 | How is your sleep situation after returning to work after giving birth? | | | | |
| 8 | What do you think is the biggest impact that returning to work after giving birth has had on you? Could you please share your thoughts? | | | | |
| 9 | Do you have any other additions? | | | | |

**Supporting Information Table 4:** Introduction to the scales and questionnaires (Quantitative).

| **Outcomes** | **Scale/Questionnaire** | **Introduction** |
| --- | --- | --- |
| 1. General Information | General demographic questionnaire | Demographic and clinical information, including age, department, work experience (years in service), body mass index (BMI), physical condition, feeding situation, and health related situation of offspring, were collected using a structured information form ^[1-2]^. |
| 2. Social support | Self-designed social support questionnaire | Social support was assessed using the version of the the perceived social support scale for nurses returning to work after childbirth tool ^[3]^. The version of the social support consists of 18 items that assess the presence of the four 4 dimensions: emotional support, appraisal support, information support, and evaluation support. The Cronbach’s alpha for the social support scale was 0.976. |
| 3. Self-efficacy | Self-efficacy questionnaire (GSE) | The general self-efficacy (GSE) scale developed by Schwarzer & Jerusalem in 1979 and was used to assess general sense of perceived self-efficacy ^[4]^. The GSE consists of 10 items. Each item refers to successful action and implies an internal/stable attribution of success with a 4-point response scale. The sum of all 10 items yields the final composite score, with a theoretical range of 10 to 40. The Cronbach’s alpha for the GSE scale was 0.85. |
| 4. Work engagement | Work engagement questionnaire (UWES-9)* | Work engagement was measured using the ultra-short version from the Utrecht Work Engagement Scale-9 (UWES-9) ^[5]^. The UWES-9 consists of 9 items.The UWES-9 assessed the three core dimensions of work engagement, with one item each: vigor, dedication, and absorption. Each dimension consists of three items. The Cronbach’s alpha for the work engagement scale was 0.93. |

***** The UWES-9 was used to measure work engagement, with a total of 9 items and a score of 0-6. The total score ranged from 0 to 54, and the average score was calculated by dividing the total score by the number of items. According to the Schaufeli and Bakker norm ^[4]^, the average score of 2.89-4.66 was the medium level (corresponding to the total score of 26.01-41.94).

**Reference**

1. Liu K, Li S, Liu Y, Yu M, Wang Y: **Breastfeeding challenges experienced by postpartum nurses after maternity leave: a qualitative systematic review**. *BMC Pregnancy Childbirth* 2026, **26**(1):178.

2. Li S, Zhang W, Liu Y, Yu M, Yang S, Luo M, Yang Q: **Lactating nurses' experiences of return to work after lifting COVID-19 lockdown: A qualitative study**. *Heliyon* 2024, **10**(1):e23761.

3. Li S, Fan J, Liu Y, Yu M, Jiang Y: **Development and psychometric properties of a perceived social support scale for nurses returning to work after childbirth**. *BMC Nurs* 2024, **23**(1):547.

4. W. B. Schaufeli, A. B. Bakker, and M. Salanova, **"The mea-surement of work engagement with a short questionnaire:a cross-national study,"** Educational and Psychological Mea-surement, vol. 66, no. 4, pp. 701-716, 2006.

5.	R. Schwarzer and A. Born, **"Optimistic self-beliefs: assessmentof general perceived self-efficacy in thirteen cultures,"** WorldPsychology, vol. 3, no. 1, pp. 177-190, 1997.

**Supporting Information Table 5**: Scores on the respective scales (Quantitative).

| Variables | Score range | M ± SD | 1 | 2 | 3 |
| --- | --- | --- | --- | --- | --- |
| **Work engagement** | 0-54 | 33.62±10.27 | - |  |  |
| Vigor | 0-18 | 10.46±3.66 |  |  |  |
| Dedication | 0-18 | 11.68±3.53 |  |  |  |
| Absorption | 0-18 | 11.48±3.71 |  |  |  |
| **Social support** | 18-90 | 74.51±11.48 | 0.702** | - |  |
| Emotional support | 4-20 | 15.78±3.07 |  |  |  |
| Instrumental support | 5-25 | 21.19±3.52 |  |  |  |
| Informational support | 5-25 | 20.65±3.90 |  |  |  |
| Appraisal support | 4-20 | 16.89±2.87 |  |  |  |
| **Self-efficacy** | 10-40 | 28.19±6.44 | 0.671** | 0.591** | - |
| **P*＜0.05; ** *P*＜0.01 | | | | | |
